# Multifractality of Pseudo-Velocities and Seismic Quiescence Associated with the Tehuantepec M8.2 EQ

**DOI:** 10.3390/e20120961

**Published:** 2018-12-13

**Authors:** Carlos Carrizales-Velazquez, Adolfo Rudolf-Navarro, Israel Reyes-Ramírez, Alejandro Muñoz-Diosdado, Lev Guzmán-Vargas, Fernando Angulo-Brown

**Affiliations:** 1 Unidad Profesional Interdisciplinaria en Ingeniería y Tecnologías Avanzadas (UPIITA), Instituto Politécnico Nacional, Ciudad de México 07340, Mexico; 2 Departamento de Física, Escuela Superior de Física y Matemáticas, Instituto Politécnico Nacional, Ciudad de México 07738, Mexico; 3 Unidad Profesional Interdisciplinaria de Biotecnología (UPIBI), Instituto Politécnico Nacional, Ciudad de México 07738, Mexico

**Keywords:** earthquake precursors, Schreider algorithm, multifractality, quiescence analysis

## Abstract

By using earthquake catalogs, previous studies have reported evidence that some changes in the spatial and temporal organization of earthquake activity are observed before and after of a main shock. These previous studies have used different approaches for detecting clustering behavior and distance-events density in order to point out the asymmetric behavior of foreshocks and aftershocks. Here, we present a statistical analysis of the seismic activity related to the $M_{w}=8.2$ earthquake that occurred on 7 September 2017 in Mexico. First, we calculated the inter-event time and distance between successive events for the period 1 January 1998 until 20 October 2017 in a circular region centered at the epicenter of the $M_{w}=8.2$ EQ. Next, we introduced the concept of pseudo-velocity as the ratio between the inter-event distance and inter-event time. A sliding window is considered to estimate some statistical features of the pseudo-velocity sequence before the main shock. Specifically, we applied the multifractal method to detect changes in the spectrum of singularities for the period before the main event on 7 September. Our results point out that the multifractality associated with the pseudo-velocities exhibits noticeable changes in the characteristics of the spectra (more narrower) for approximately three years, from 2013 until 2016, which is preceded and followed by periods with wider spectra. On the other hand, we present an analysis of patterns of seismic quiescence before the $M_{w}=8.2$ earthquake based on the Schreider algorithm over a period of 27 years. We report the existence of an important period of seismic quietude, for six to seven years, from 2008 to 2015 approximately, known as the alpha stage, and a beta stage of resumption of seismic activity, with a duration of approximately three years until the occurrence of the great earthquake of magnitude $M_{w}=8.2$. Our results are in general concordance with previous results reported for statistics based on magnitude temporal sequences.

## 1. Introduction

Diverse methods from complexity studies have been applied to evaluate spatio-temporal organization of seismic activity from different regions of the world. An important aspect of some of these studies is that they intend to identify potential anomalous patterns prior to the occurrence of a mainshock. Although there is some debate about the universality of these features, there are efforts to elucidate the dominance of some peculiar spatio-temporal organizational patterns in specific regions with high seismic activity. The occurrence of earthquakes is a complex phenomenon which results from interactions covering a wide range of spatio-temporal scales, with some statistical properties frequently expressed as power-law functions with fractal behavior. In many cases, the use of a single scaling exponent is not sufficient to fully characterize the variety of dynamical behaviors displayed by earthquake activity, and the use of multifractality is necessary, where a broad distribution of fractal scaling exponents is associated with the richness of the dynamics. For instance, a multifractal structure of inter-event time series has been reported for various earthquake sequences [1,2,3,4,5,6,7]. However, it is widely accepted that the complexity of earthquake activity is observed in both spatial and temporal domains, the use of parameters which contain information from both sides being necessary. This is compatible, for example, with the findings of the analysis in natural time [8] of the seismicity in Japan for 1994–2011, which revealed that the spatiotemporal variations of the order parameter of seismicity enable the estimation of the epicentral location of forthcoming major earthquakes [9].

In a related way, the identification of a seismic quiescence, defined as a temporal window where a function associated with the rate of seismic process attains an abnormal value, represents a potential source of information to characterize anomalous seismic activity [10]. Probably the seismic quiescence is the most reported seismicity pattern, prior to the occurrence of a large earthquake [11,12]. The Mexican coast of the Pacific is not the exception; for the last five decades, four earthquakes of magnitude $M_{w}>7.7$ have been preceded by important patterns of seismic quiescence [13,14]. As we said, on 7 September 2017, a great earthquake of $M_{w}=8.2$ occurred at Tehuantepec Isthmus in Mexico. For a long time, some seismologists thought that in this section of the Pacific trench, an aseismic slip of the Cocos plate could occur or a recurrence period for big earthquakes could be abnormally large [15]. However, the great earthquake of 7 September 2017 helps to better understand the dynamics of the subduction process [16,17]. Motivated by this event, we decided to investigate if this great earthquake had some particular statistical features and precursor quietude patterns similar to those we have reported previously [14]. We report that the spatio-temporal organization represented by the pseudo-velocities exhibits changes in the multifractality with a narrower and asymmetric spectrum for the years 2013 until almost the end of 2016. We also found that the Schreider algorithm shows an important period of seismic quietude, for six to seven years, from 2008 to 2015 approximately, known as the alpha stage, and a beta stage of resumption of seismic activity, with a duration of approximately three years until the occurrence of the great earthquake of magnitude $M_{w}=8.2$, occurred on 7 September 2017, with an epicenter in the region of the Tehuantepec Isthmus in Mexico. The alpha and beta stages are taken in the sense of Ohtake [18]. The paper is organized as follows. In Section 2, a description of the dataset and methods is presented. The results and discussion are described in Section 3. Finally, some conclusions are given in Section 4.

## 2. Data and Methods

### 2.1. Seismic Data and Pseudo-Velocities

**Figure 1:**
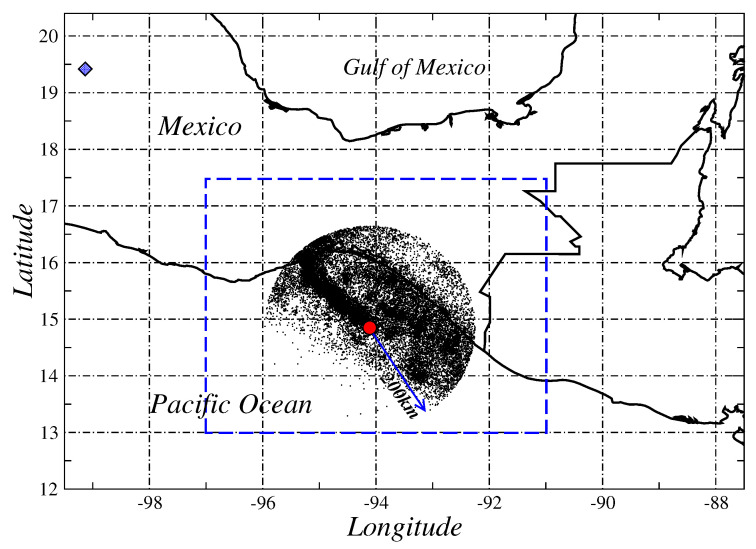
Earthquake activity of Tehuatepec region for the period January 1998 until June 2018. We show the seismic activity within a circle of radius of 200 km of the $M_{w}=8.2$ earthquake (red point) that occurred on 7 September 2017. This event was located 745 km from Mexico City (blue diamond). We also show a rectangle for which we determine the completeness magnitude of the used catalog.

The information of seismic activity was directly obtained from the *Servicio Sismológico Nacional* (*SSN*), *Universidad Nacional Autónoma de México* (www.ssn.unam.mx). Our dataset contains events for the period January 1998 until 12 June 2018. The geographical region under study is defined by a circle of radius R=200 km centered at the epicenter of the $M_{w}=8.2$ with coordinates 14.761 N and 94.103 W (see Figure 1). We notice that, for the period of observation, the catalog contains different minimum values for magnitudes; then, we restrict ourselves to analyze events with magnitude greater or equal than 3.8; this is the magnitude for which our catalog is complete for the rectangle shown in Figure 1.

**Figure 2:**
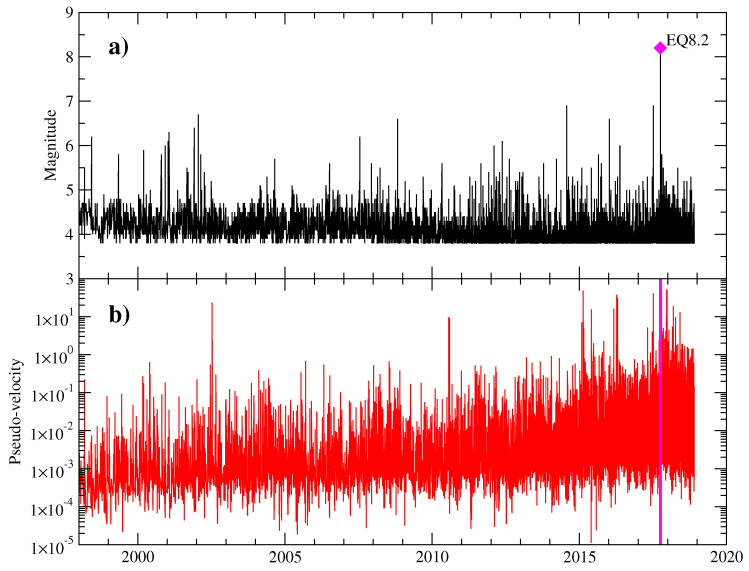
(**a**) sequence of earthquake magnitudes from Tehuantepec region; (**b**) time evolution of pseudo-velocities (Δr/Δt) obtained from seismic activity shown in (**a**).

We define the pseudo-velocity of a “walker” between two consecutive events as $v_{w}=\log\left(\frac{\Delta r}{\Delta t}\right)$, where Δr is the distance between two consecutive earthquakes and Δt the time between them. In what follows, we shall ignore the vectorial character of the velocities and focus on the temporal organization of their values. Figure 2a,b shows the sequence of magnitudes and time evolution of the pseudo-velocities for the period under observation.

### 2.2. Multifractal Detrended Fluctuation Analysis

The multifractal detrended fluctuation analysis (MDFA) was intruduced by Kantelhardt et al. [19] as an extension of the monofractal DFA method [20], which is a reliable method to detect the presence of correlations in irregular time series. Briefly, we explain the main steps of the MDFA. Consider the time series $X_{i}$ of length *N*. First, an integration is performed,

(1) $$Y_{i}=\sum_{k=1}^{i}\left(X_{k}-\bar{X}\right),\;i=1,2,3,\ldots ,N,$$

where $\bar{X}$ represents the mean value. Next, the integrated time series is divided into $N_{s}=\left\lfloor N/s\right\rfloor$ non-overlapping segments of equal size *s* (where ⌊.⌋ is the floor function). The same procedure for the backward time series is considered. Thus, $2N_{s}$ segments are obtained and the trend within each segment is calculated by means of a polynomial fit. The variance for each segment ν is given by

(2) $$F\left(s,\nu \right)=\frac{1}{s}\sum_{i=1}^{s}Y_{(\nu -1)s+i}-y_{i}^{\nu },$$

where $y_{i}^{\nu }$ is the fit of the ν-th segment. After detrending the time series, an average is taken to get the *qth-order* fluctuation function given by

(3) $$F_{q}\left(s\right)={\left[\frac{1}{2N_{s}}\sum_{\nu =1}^{2N_{s}}{\left[F\left(s,\nu \right)\right]}^{q/2}\right]}^{1/q},\;q\in Z.$$

If the original time series exhibits multifractal properties, $F_{q}\left(s\right)$ will follow a power-law of the form,

(4) $$F_{q}\left(s\right)∼s^{h_{q}}.$$

The exponent $h_{q}$ has a linear relation with the well-known mass exponent $\tau_{q}$ of the multifractal formalism, $\tau =qh_{q}-1$ [19,21]. Finally, the multifractal spectrum f(α) is obtained by means of the Legendre transformation [19,21]:

(5) $$\alpha_{q}=\frac{d\tau_{q}}{dq}\;\mathrm{and}\;f\left(\alpha \right)=q\alpha_{q}-\tau_{q}.$$

Here, α represents the *singularity strength* or *Hölder exponent* and f(α) denotes the fractal dimension of the subsets of the series characterized by α [19]. The characterization of the multifractal spectrum is given by its width $\Delta \alpha =\alpha_{max}-\alpha_{min}$, the $\alpha^{*}$-value which corresponds to the maximum value of f(α) and the asymmetry parameters $\alpha_{right}=\alpha_{max}-\alpha^{*}$ and $\alpha_{left}=\alpha^{*}-\alpha_{min}$.

### 2.3. Schreider Algorithm

We assume that the *SSN* catalog is reliable and complete for the application of the Schreider algorithm [10]. A magnitude $M_{o}$ as the minimum threshold is chosen. First, we consider cylindrical volumes of variable radius and height, centered in the hypocenter of the Tehuantepec earthquake, in order to search for patterns of seismic quiescence in a temporal space interval and within a given range of magnitudes. Then, we calculate the function

(6) $$T^{\prime }\left(n\right)=t\left(n\right)-t\left(n-1\right),$$

where t(n) is the occurrence time of the *n*-th earthquake inside the cylindrical volume, with magnitude $M\geq M_{o}$. The function $T^{\prime }$ is the time between consecutive events that can be assigned to each point of the seismic region; small values of $T^{\prime }$ indicate that earthquakes occur frequently, and big values of $T^{\prime }$ correspond to low seismic activity. A smoothness procedure is applied calculating the convolution function of $T^{\prime }$ by means of the Laplace function [10],

(7) $$f\left(n,s\right)=\frac{1}{s\sqrt{2\pi }}\exp\left[-\left(n^{2}/2s^{2}\right)\right],\;n=1,2,3,\ldots ,$$

where *s* is the *smoothness* parameter [10]. Therefore, each *k*-th seismic event is related to the convolution

(8) $$T\left(k\right)=\sum_{n=0}^{l}T^{\prime }\left(k-n\right)f\left(n,s\right).$$

The limit *l* is determined when the function f(n,s) is approximately zero. To identify a period of seismic quiescence, it is necessary to know the average value of T(k) which is calculated in an interval of at least two decades (we consider that this value is stable for this period of time). The average of T(k) is given by

(9) $$\bar{T}=\frac{1}{N}\sum_{k=1}^{N}T\left(k\right),$$

where *N* is the total number of earthquakes with $M\geq M_{o}$ inside the cylindrical volume of exploration. The standard deviation is calculated as

(10) $$\sigma =\sqrt{\frac{\sum_{k=1}^{N}{\left[T\left(k\right)-\bar{T}\right]}^{2}}{N-1}}.$$

Schreider considers that the temporal convolution function T(k) has a Gaussian distribution; then, values of $T\left(k\right)\geq \bar{T}\pm 3\sigma$ are considered abnormally big; therefore, we can say that a point with coordinates (ϕ,λ) (the center of the exploration cylinder) presents an abnormal seismic quiescence if $T\left(k\right)\geq \bar{T}\pm 3\sigma$.

## 3. Multifractality of Pseudo-Velocities

**Figure 3:**
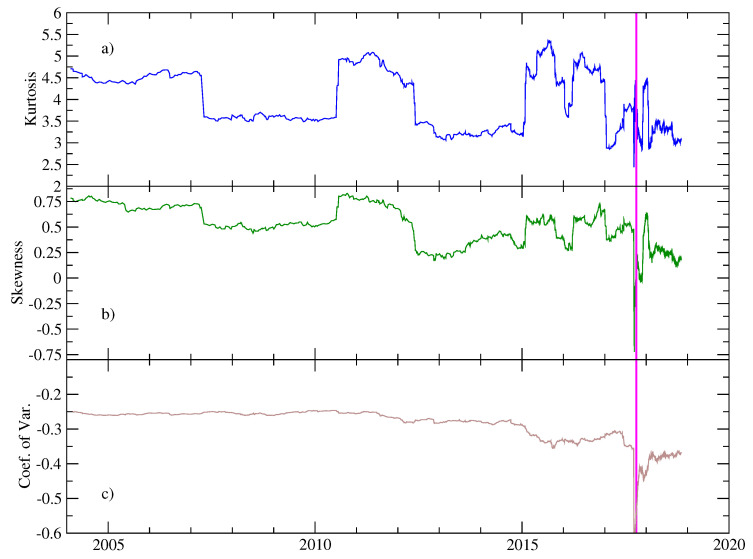
Statistical parameters of pseudo-velocities as a function of time. We consider a sliding window with $10^{3}$ values and an overlapping of 990 data points. For each window, we calculated (**a**) the kurtosis, (**b**) the skewness and (**c**) the coefficient of variation.

Prior to the description of multifractal properties of the pseudo-velocities, we describe some representative statistical parameters of the corresponding velocity distributions along the time. Specifically, a sliding window which contains $10^{3}$ data values is considered with an overlapping of 990 values. For each window, we calculated the usual statistical parameters: the skewness $S_{k}$, the Kurtosis *K*, and the coefficient of variation *C*. $S_{k}$ is a measure of the lack of symmetry. Negative (positive) values are associated with the existence of left (right) asymmetric tails longer than the right (left) tail. *K* is a measure of whether the data are peaked (*K* >3) or flat (K<3) relative to a normal distribution. The coefficient of variation is defined as the ratio between the standard deviation and the mean value. Figure 3a–c shows the results of these metrics as a function of time for the period before the main shock. We observe that $S_{k}$ is positive along the whole period, starting from 1 with fluctuations around 0.5, indicating that distributions are positively skewed. There are some periods for which $S_{k}$ is close to the neutral zero value. We will discuss this property later in the Results section. After the EQ8.2, $S_{k}$ is also close to zero, indicating that pseudo-velocities are well described by a normal distribution. For the Kurtosis *K*, the evolution displays values between 3 and 5, denoting that pseudo-velocities distributions are relatively more peaked compared to the Gaussian distribution. We roughly identify several periods for which *K* has a lower local value, revealing that, for these windows, the distributions are identified as quasi-symmetrical and close to the Gaussian one. The coefficient of variation exhibits a slowly decreasing behavior with some fluctuations until the occurrence of the main shock. This behavior is expected due to the fact that the mean value decreases for the whole interval before the mainshock (see Figure 3c).

**Figure 4:**
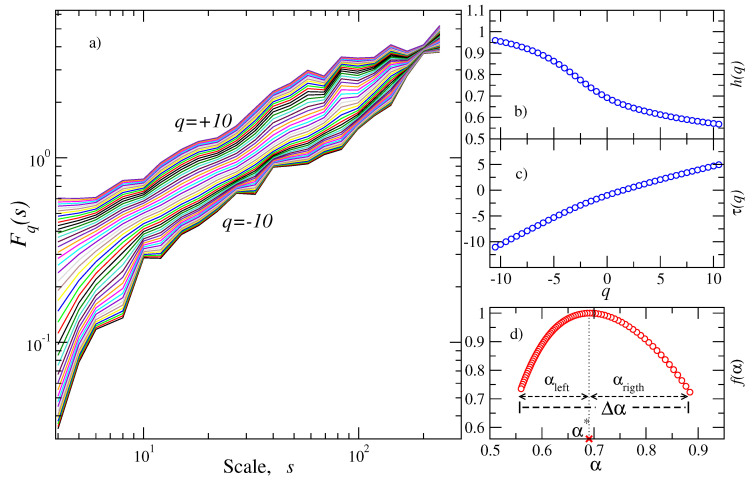
Multifractal detrended fluctuation analysis (MDFA) of a representative segment of pseudo-velocities. (**a**) log-log plot of $F_{q}\left(s\right)$ vs. *s* for several values of *q*; (**b**) $h_{q}$ vs. *q* for the data in (**a**); (**c**) exponent τ(q) vs. *q* for the data in (**a**); (**d**) multifractal spectrum f(α) vs. α.

**Figure 5:**
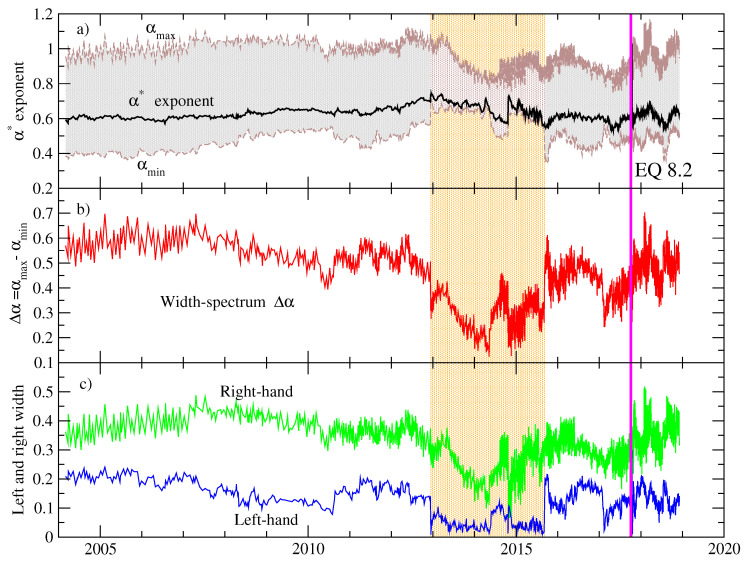
Time evolution of representative multifractal parameters. (**a**) the exponent $\alpha^{*}$, $\alpha_{max}$ and $\alpha_{min}$; (**b**) width of the multifractal spectrum; (**c**) asymmetry parameters: right-hand and left-hand width.

Next, we applied the MDFA method to the same windows (with $10^{3}$ values) as described above. Figure 4a shows representative cases of the spectra $F_{q}\left(s\right)$ vs. *s* for several values of *q* within the interval [−10,10]. The estimation of the scaling exponents $h_{q}$ (and τ(q)) permits determining the dependence of these exponents in terms of the *q*-moments as shown in Figure 4b,c, and, finally, the multifractal spectrum is constructed (see Figure 4d). We focused our attention on the evolution of the parameters which characterize the multifractality within each window. The results are depicted in Figure 5. The exponent $\alpha^{*}$ exhibits a slight upward trend from 0.6 to 0.7 for the period 2004 until the end of 2012 (see Figure 5a); from the beginning of 2013 until approximately the end of 2015, the $\alpha^{*}$-exponent fluctuates between 0.6 and 0.7 and then fluctuations around 0.6 are observed until the main shock occurred. Interestingly, a noticeable decrease in the width of the spectrum (Δα≈0.3) is clearly observed for the period 2013 until the end of 2015 (vertical shaded area shown in Figure 5a), suggesting that a more monofractal behavior is present for this period (see Figure 5b). Outside this interval, the width of the spectrum exhibits higher values ([0.4,0.7] before 2013 and [0.3,0.6] after 2015) until the mainshock, revealing a broad multifractality. Regarding the asymmetry parameters (see Figure 5c), the left-hand width that corresponds to positive moments (large fluctuations) suffers the most noticeable reduction for the period where the width is narrower, while, for the right-hand width (small fluctuations), the reduction is smaller. This indicates that large fluctuations are the ones that contribute to the reduction of the spectrum. For the periods outside this region, the asymmetry parameters are higher except for a short interval just before the main shock.

**Figure 6:**
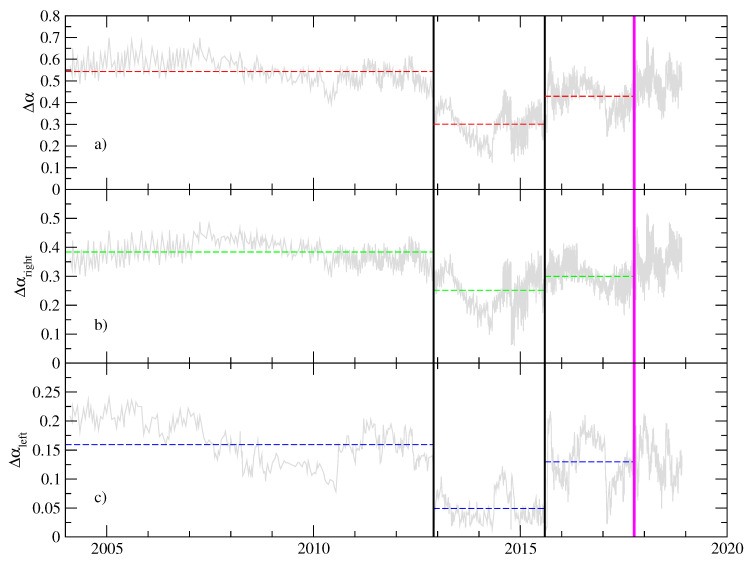
Segmentation results of the representative multifractal parameters from pseudo-velocity time series. The method leads to partitioning of the sequence into intervals with means, each significantly different from the mean of the adjacent segments. The significance level is 0.95. In our case, we stop the process after the second iteration, leading to three segments depicted in panels (**a**–**c**).

In order to provide a statistical assessment of the differences between the periods described above, we apply a segmentation method based on significant differences between the mean values of adjacent segments in a non-stationary sequence [22]. The method is able to separate two segments with a statistically different mean. A significance level is applied to cut the series into two new segments (typically set to 0.9), as long as the means of the two new segments are significantly different from the mean of the adjacent segments (see details in [22,23]). The segmentation procedure was applied to the multifractal parameters: Δα, $\Delta \alpha_{right}$ and $\Delta \alpha_{left}$. The results are shown in Figure 6. Clearly, the segmentation procedure separates the three regions previously identified by inspection (see Figure 5), confirming that the mean values of the multifractal parameters of adjacent segments are significantly different. We remark that the segment which corresponds to the period with a narrower spectrum, roughly also corresponds to the interval for which both the kurtosis and the skewness were concomitant with a Gaussian distribution (see Figure 3).

## 4. Quiescence Analysis

**Figure 7:**
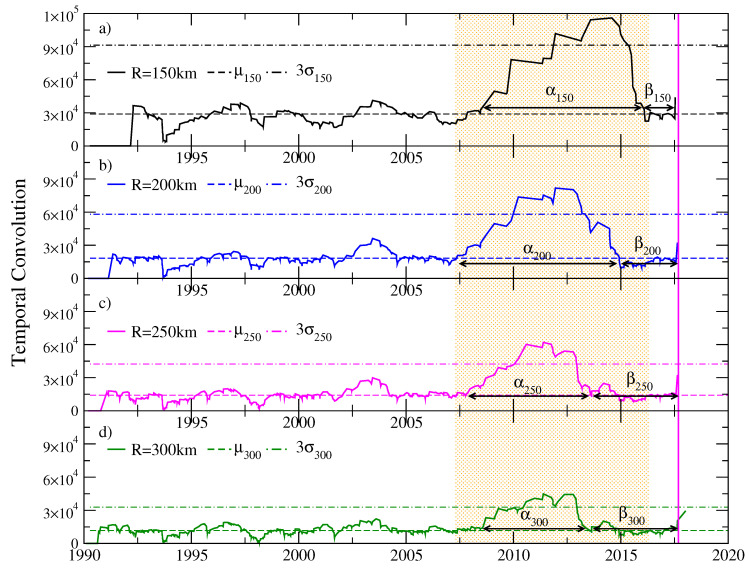
Temporal convolution function for earthquakes in the region of the Isthmus of Tehuantepec. Cylindrical exploration volumes with different radii are considered: (**a**) R=150 km, (**b**) R=200 km, (**c**) R=250 km and (**d**) R=300 km. The periods of seismic stillness (alpha stage) and resumption of seismic activity (beta stage) depend on the radius of the exploration cylinder.

**Figure 8:**
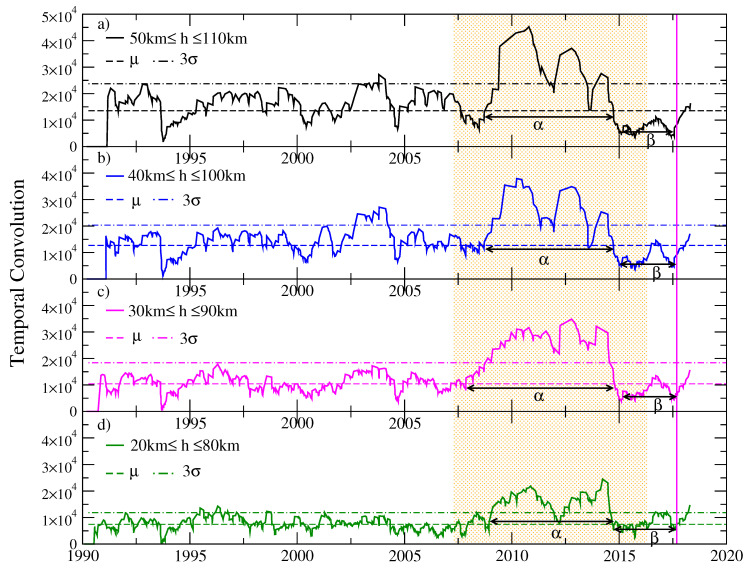
Temporal convolution of earthquakes with coda threshold magnitude $M_{o}\geq 4.3$ within different cylindrical volumes centered at the epicenter of the Tehuantepec earthquake, $M_{w}=8.2$, with a radius of 200 km and a height of 60 km. The depth of the exploration cylinders are: (**a**) h=50 km, (**b**) h=40 km, (**c**) h=30 km and (**d**) h=20 km. The smoothing parameter used is s=9; with this parameter, the beginning and end of the alpha stage is better appreciated.

**Figure 9:**
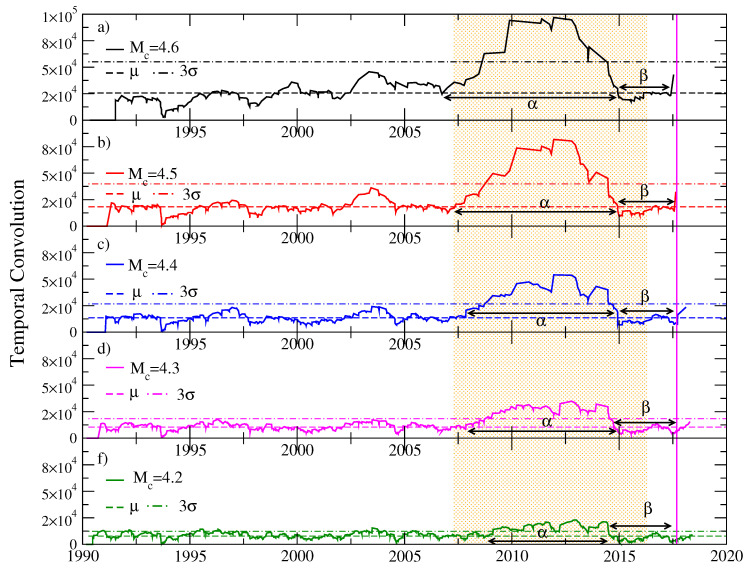
Temporal convolution of earthquakes with coda threshold magnitude (**a**) $M_{c}=4.6$, (**b**) $M_{c}=\;4.5$, (**c**) $M_{c}=4.4$, (**d**) $M_{c}=4.3$ and (**d**) $M_{c}=4.2$, for a cylindrical volume centered at the epicenter of the Tehuantepec earthquake, with a radius of 200 km and a height of 60 km. The depth of the events varies between 30 and 90 km. The smoothing parameter used is s=9.

We applied the Schreider algorithm for earthquakes of coda magnitude $M_{c}\geq M_{o}$ that occurred between January 1990 and September 2017 in the region of Tehuantepec Isthmus, considering a cylindrical volume of exploration centered in the epicenter of the earthquake of magnitude $M_{w}=8.2$, latitude 14.761 N, and longitude −94.103 W. We considered an interval of 60 km for the depth of the events inside the exploration cylinder, which we were moving down and found an important episode of seismic quiescence for events between 30 and 90 km deep. In each of Figure 7, Figure 8 and Figure 9, horizontal dotted lines represent the average of each time series and the value $\bar{T}+3\sigma$. We will call *Schreider’s critical value* the value of $\bar{T}+3\sigma$, given that values of the convolution T(k) that exceed this critical value are considered abnormally large.

Figure 7 shows four temporal convolution functions for earthquakes of magnitude $M_{c}\geq 4.5$ occurring between January 1990 and September 2017. The exploration cylinders for the calculation of the convolution function considered events with depths between 30 and 90 km. The graphs correspond to cylinders with different radii: R=150 km (black line), R=200 km (blue line), R=250 km (magenta line) and R=300 km (green line). In Figure 7a–d, it can be seen that the duration of the alpha and beta stages depend on the radius of the exploration cylinder; these figures allow us to see that, for a radius of 200 km, the temporal convolution function acquires the highest values within the quiet period. For this reason, the following analyses were made keeping that radius of the cylinder.

Figure 8 shows four temporal convolution functions for earthquakes of magnitude $M_{c}\geq 4.3$, for different exploration cylinders with 200 km radius and 60 km height, whose depth of the upper face varies between 20 and 50 km deep. This figure shows the temporal convolution function for different depths of the exploration cylinders. We observe that, at the beginning of 2009, a significant increase in the value of the convolution function began, exceeding the critical value of Schreider between the beginning of 2010 and the end of 2014, which we consider as the alpha stage. At the beginning of 2015, the beta stage of resumption of seismic activity can be seen, with values below the average, which indicates a period of important activity, until the occurrence of the earthquake on 7 September 2017. It is noted, for a depth of events between 30 and 90 km, that the function of temporal convolution is maintained, within the quiet period (alpha stage), with the highest number of values above Schreider’s critical value; for this reason, in the following analysis, interval of depths is considered.

In Figure 9, we have five temporal convolution graphs, evaluated for a cylindrical volume of 200 km radius between 30 and 90 km depth and centered at the epicenter of the Tehuantepec earthquake, for different values of the threshold coda magnitude 4.2≤4.6. Different alpha and beta stages are observed, for each magnitude whose duration varies between six years for threshold magnitude $M_{c}=4.2$ and eight years for $M_{c}=4.6$. The duration of the beta stage is approximately three years. The durations of both of these stages, alpha and beta, are consistent with the results obtained from the application of natural time analysis (e.g., see Ref. [24] as follows: Ramirez-Rojas and Flores-Marquez [25] studied the fluctuations of the order parameter of seismicity by employing natural time analysis of seismicity for the period 1974–2012 in six tectonic regions of the Mexican Pacific coast and identified the following two key properties in the Chiapas region discussed later by Sarlis et al. [26]: first, the probability density of the order parameter fluctuations had a bimodal feature. Second, the feature of the scaled distribution was non-Gaussian having a left exponential tail. These two features that have been identified almost five years before the occurrence of the M8.2 earthquake on 7 September 2017 signaled a forthcoming large earthquake. In addition, Ramirez-Rojas et al. [27] using sliding windows comprising $4\times 10^{3}$ and $5\times 10^{3}$ events of magnitude 3.5 or larger identified in natural time important precursory variations in the entropy change under time reversal.

## 5. Conclusions

We have presented results of multifractal properties of pseudo-velocities and quescience obtained from spatio-temporal information of the 1998–2017 earthquake activity in the Tehuantepec region. Our results of multifractality revealed that a reduction in the width of the spectrum is observed for the years 2013–2015, indicating that, for this interval, the pseudo-velocity exhibited a less multifractal (more monofractal) behavior with a notorious reduction of the left-hand width of the spectrum for a period that comprises the beginning of 2013 until the end of 2015. The monofractality (narrow spectrum) indicates that the pseudo-velocity sequence exhibits less nonlinear features compared to a broad multifractal spectrum. Moreover, the Schreider algorithm was used for the identification of the pattern of significant seismic quietude within a cylindrical volume of a 200 km radius, located between 30 and 90 km depth and centered at the epicenter of the earthquake of magnitude $M_{w}=8.2$. The analysis of the temporal series of earthquakes that occurred between January 1990 and September 2017, in the Tehuantepec region, considering a range of threshold magnitudes $4.2<M_{c}<4.7$ for the application of the algorithm, shows a pattern of significant seismic stillness that begins in the year 2008 and ends at the end of 2015, a temporary interval of approximately seven years (alpha stage), which ends with the resumption of seismic activity in the region, activity superior to the average historical activity of the last three decades (beta stage) that lasts until the occurrence of the main event of magnitude $M_{w}=8.2$. This pattern of seismic stillness is similar to those reported by [14] for earthquakes occurring in the Mexican subduction zone of the Pacific with $M_{w}\geq 7.8$. Finally, we remark that the two methods used in our study identified significant patterns from spatio-temporal information of earthquake activity for approximately the same period before the main shock, and additional studies are needed in this direction.
